# Medication adherence among Japanese patients with developmental disabilities: a survey study

**DOI:** 10.3389/fpsyt.2024.1431604

**Published:** 2024-10-21

**Authors:** Mutsumi Ando, Iori Taki, Taigi Yamazaki, Noriko Hida

**Affiliations:** ^1^ Division of Clinical Research and Development, Graduate School of Pharmacy, Showa University, Setagaya-ku, Japan; ^2^ Department of Clinical Pharmacy, Division of Clinical Research and Development, School of Pharmacy, Showa University, Setagaya-ku, Japan

**Keywords:** developmental disabilities, pharmacotherapy, medication adherence, medication instructions, medication record book

## Abstract

**Aim:**

Developmental disabilities (DD) often persist into adulthood, necessitating early and continuous treatment. Although pharmacotherapy is a viable treatment option, managing medication can be challenging. Prior research has not explored medication use among patients with DD in Japan. Thus, this study aimed to identify the medication challenges faced by these patients.

**Methods:**

A questionnaire survey was administered to 200 outpatients to collect data on the number of prescribed medications, the timing of administration, the frequency of missed doses, and the use of medication notebooks. This was a prospective observational study without intervention and a random sampling.

**Results:**

The survey revealed that 57.0% of the participants were non-adherent to their medication regimen. Specifically, medication non-adherence rates were 44.7% among individuals with autism spectrum disorder (ASD), 86.9% for those with attention deficit hyperactivity disorder (ADHD), and 30.4% for patients with comorbid ASD and ADHD. Despite 48.3% of the participants taking measures to prevent forgetting their medication, 65.3% of them still failed to take their medication as prescribed. The possession rate of medication record books was 96.0%.

**Conclusion:**

The study indicates that the frequency of medication non-adherence among patients with DD in Japan mirrors that in other countries. Patients who reported taking preventative measures still experienced high rates of non-adherence, suggesting limited effectiveness of these strategies. It is essential to develop more effective measures to improve adherence, enhance disease awareness, and increase understanding of medication instructions. The high possession rate of medication record books suggests they could play a significant role in managing DD, and their use is expected to increase in the future.

## 1 Introduction

Developmental disabilities (DD) typically affect individuals from birth, influencing their physical, intellectual, and emotional development (1–3). DD may persist into adulthood (2, 4, 5). Attention deficit hyperactivity disorder (ADHD) is thought to arise from a genetic predisposition and neuropsychological dysregulation (6), which can vary by age and developmental stage relevance (7).

The World Mental Health Survey reported that the average prevalence of ADHD in adults, as defined by the Diagnostic Statistical Manual of Mental Disorders Fourth Edition and the Composite International Diagnostic Interview, was 2.8% in 2017 (5). Psychiatric disorders might emerge before the first distinct symptoms of ADHD manifest. Furthermore, psychiatric disorders often coexist with clinically significant levels of ADHD symptoms (8). Most comorbidities appear subsequent to the onset of ADHD in patients with psychiatric disorders (2, 8). Among adults with DD, the prevalence of autism spectrum disorder (ASD) is approximately 1% (9, 10). Studies have shown that 65% to 85% of individuals with ASD experience psychiatric comorbidities (11, 12). The challenging behaviors in adults with ASD and intellectual disability intensify with symptom severity, underscoring the need for lifelong intervention programs (13).

The challenges associated with medication adherence in patients with DD can be examined from three distinct perspectives: cognitive function, behavioral/emotional, and communication (14). Patients with DD may experience difficulties in recalling the name of their medication, the time at which it should be taken, and the correct method of administration (15). Such difficulties are frequently attributable to deficiencies in cognitive functions, including memory, attention, and organizational abilities (15). The use of visual reminders and alert systems, straightforward instructions, and verbal prompts has been demonstrated to be an effective method for addressing these issues (15). Furthermore, patients with DD may also experience difficulty in accepting new routines or in complying with medication regimens. Such difficulties are frequently attributable to shortcomings in self-control and the capacity to regulate emotions (16). Behavioral therapy, counseling, and pharmacotherapy have been demonstrated to be effective in overcoming these problems (14). In addition, patients with DD may encounter difficulties in communicating with medical professionals, which can impede their understanding of the importance of medication and its proper administration (14). This is frequently attributed to challenges in language proficiency and social skills (14). Speech therapy, social skills training, and the support of family and caregivers can effectively address these issues (14, 15). These challenges can potentially lead to reduced medication adherence and diminished treatment efficacy (16). Therefore, it is crucial to comprehend these concerns and provide suitable assistance.

Early and sustained interventions, such as medication, specialized education, speech and communication therapy, and behavioral modifications are essential for patients with DD (14). Therefore, therapeutic responses are critical. However, unsuccessful medication use may result from the medication itself or issues with medication adherence (16). In a questionnaire survey, over 35% of support workers cited “lack of clear results from medication” and “self-discontinuation of medication” as challenges among adults with ASD (16). An analysis of open-ended responses using the KJ method identified common difficulties faced by support workers in medication management, including “difficulty in conveying the necessity for medication” and “challenges in managing medication routines” (16).

Moreover, pharmacotherapy is vital in the long-term management of ADHD, with treatments including noradrenergic agonists, α-agonists, and antidepressants (17, 18). It is noteworthy that while few disorders among patients with DD are curable, existing treatments can significantly alleviate core symptoms in patients with ADHD (11). Factors influencing medication adherence include societal attitudes towards drug use, pressure, concerns, and inconvenience (19). Non-adherence rates for stimulants used in treating ADHD, such as methylphenidate and amphetamines, range from 13.2% to 65% (19, 20).

Discontinuing medication for ADHD may result in increased behavioral issues, anger, and frustration, adversely affecting life at home, work, and school (21). The National Institute for Health and Care Excellence (NICE) guidelines for ADHD mandate that individuals with ADHD assume responsibility for their health, which includes adhering to prescribed medications (15). Clear instructions regarding medication usage, covering dosage, duration, adverse effects, and scheduling, should be provided in written or pictorial form and accompany the medication (e.g., a sticker on the side of the packet) (15). Utilizing visual aids such as apps, alarms, clocks, pill dispensers, or notes on calendars or refrigerators can facilitate regular medication intake (15). Incorporating medication into daily routines, such as taking it before meals or after brushing teeth, is also beneficial (15). Peer support groups for individuals with ADHD, their families, and caregivers can be advantageous (15). Additionally, it is important for parents and caregivers to supervise the administration of ADHD medications to children and adolescents (15).

In Japan, healthcare providers can enhance patient adherence by offering medication guidance through pharmacists (22) and employing visually engaging tools that incorporate patient feedback (23, 24). Medication record books—booklets that document a patient’s medication history chronologically and serve as support tools for managing treatment (25)—have shown to improve medication adherence among older adults in Japan (26). However, it remains uncertain which strategies patients with DD employ to manage their medication.

It is essential to develop more effective measures to improve adherence, enhance disease awareness, and increase understanding of medication instructions. However, prior research has not explored medication use among patients with DD in Japan. First of all, this study aimed to identify the medication challenges faced by these patients. Thus, this study sought to elucidate the medication management challenges faced by patients with DD who were seen at the Showa University Medical Institute of Developmental Disabilities Research. The insights gained will assist in identifying support mechanisms to enhance medication adherence.

## 2 Methods

### 2.1 Participants and methods

This clinical trial was conducted at the Showa University Medical Institute of Developmental Disabilities Research and received approval from the Showa University Research Ethics Review Board (22-164-B). This was a prospective observational study without intervention and a random sampling. After the researcher provided an explanation, the participants were asked to indicate their consent for participation in the study by checking the box labeled “I agree to participate in the study” on the questionnaire. The study included 200 participants who were directly informed about this study by the researchers in person, consented to participate, and met the eligibility criteria. The research was carried out from January to June 2023. A questionnaire survey was administered using Google Forms to assess the medication status of patients with DD who agreed to participate.

Inclusion criteria encompassed participants who were: 1) outpatients at the Showa University Medical Institute of Developmental Disabilities Research, 2) individuals 18 years of age or older at the time consent was obtained, and 3) patients who consented to participate in the study. Participants who faced challenges completing the questionnaire were excluded based on the investigator’s discretion. This study was conducted following the Declaration of Helsinki and the Ethical Guidelines for Medical Research Involving Human Subjects.

### 2.2 Survey items

The questionnaire administered to patients collected data on the following variables. This questionnaire was specifically developed for this study: 1) patient characteristics, including age, sex, diseases, time of onset, family history, length and frequency of hospital visits, and educational history; 2) medication history, which detailed the number of medications, number of doses, and timing of administration; 3) medication adherence, encompassing the frequency of missed doses, timing of recall for missed doses, and strategies employed to enhance medication compliance; Regarding the frequency of forgetting to take medication, the options were “often,’’ “occasionally,’’ “rarely,’’ and “never.’’ Patients who answered “often’’ or “occasionally’’ were defined as having medication non-adherence. 4) utilization of a medication record book, which noted its presence or absence, the percentage of participants bringing it, the extent of its use, and reasons for its use. Regarding the use of medication notebooks, the options were “strongly agree,’’ “somewhat agree,’’ “do not strongly agree,’’ and “do not agree.’’ Patients who answered “strongly agree” or “somewhat agree” were identified as effectively utilizing medication record books. Those who answered “do not strongly agree” or “do not agree” were viewed as not effectively utilizing them.

### 2.3 Statistical analysis

The data from all respondents who completed the survey were aggregated in a descriptive manner using Excel and JMP ^®^ Pro 16.0.0 (SAS, Tokyo, Japan). For each survey item, the total responses for each option were calculated, and percentages were derived.

## 3 Results

### 3.1 Patient characteristics

This study involved 200 patients at the Showa University Medical Institute of Developmental Disabilities Research during the survey period. Two patients were excluded: one who declined consent and another who provided duplicate responses. Consequently, data from 198 patients were analyzed.

**Table 1** details the demographic backgrounds of the participants. The age at which consultation was sought for the identified condition was recorded as the age of onset. In this study, 55.1% of patients reported that they were 18 years of age or older when they first visited a doctor. There were 125 patients with vocational school, university, or graduate school education. By disorder, the largest number of patients with ASD was 65 (32.8%), followed by 55 patients (27.8%) with ADHD.

**Table 1:** Patient characteristics (n = 198).

Sex, n (%)	
Male	114 (57.6)
Female	81 (40.9)
Do not answer	3 (1.5)
Age (years), median (IQR)	33 (26-42)
Age of onset (year), n (%)	
< 18	40 (20.2)
≧ 18	109 (55.1)
Missing responses	49 (24.7)
Final education, n (%)	
Junior high school	22 (11.1)
High school	53 (26.8)
Vocational school	24 (12.1)
University	90 (45.5)
Graduate school (master’s degrees)	9 (4.5)
Graduate school (doctoral degrees)	0 (0.0)
Disorders, n (%)	
no other disorders	
ASD†	65 (32.8)
ADHD‡	55 (27.8)
Other disorders§	30 (15.2)
with other disorders	
ASD + ADHD	30 (15.2)
ASD + ADHD + Other disorders¶	6 (3.2)
ASD + Others	4 (2.0)
ADHD + Other disorders¶	8 (4.0)

†ASD, Autism Spectrum Disorder.

‡ADHD, Attention Deficit Hyperactivity Disorder.

§Other disorders: Learning Disability(LD), Stuttering, Others.

¶Other disorders: LD, Tic disorders, Others.

### 3.2 Medication history

The study found that 149 patients (75.3%) were on medication.

**Table 2** displays the number of medications, number of doses, and timing of administration. The most common number of medications taken was 1-3, with 89 patients taking them. Additionally, 68 patients took their medications once a day, and 83 patients took their medications before bedtime.

**Table 2:** Information about medications being taken (n = 149).

Number of medications, n (%)		Number of doses, n (%)		Timing of administration (multiple choice), n	
1-3	89 (59.7)	Once daily	68 (45.6)	When you wake up	22
4-5	32 (21.5)	Twice daily	40 (26.8)	Before breakfast	21
6-7	14 (9.4)	Thrice daily	29 (19.5)	After breakfast	64
8-9	8 (5.4)	Four or more times daily	12 (8.1)	Before lunch	10
≧ 10	6 (4.0)			After lunch	24
				Before dinner	14
				After dinner	72
				Before bedtime	83
				As-needed medication	21

### 3.3 Medication adherence

**Figure 1:**
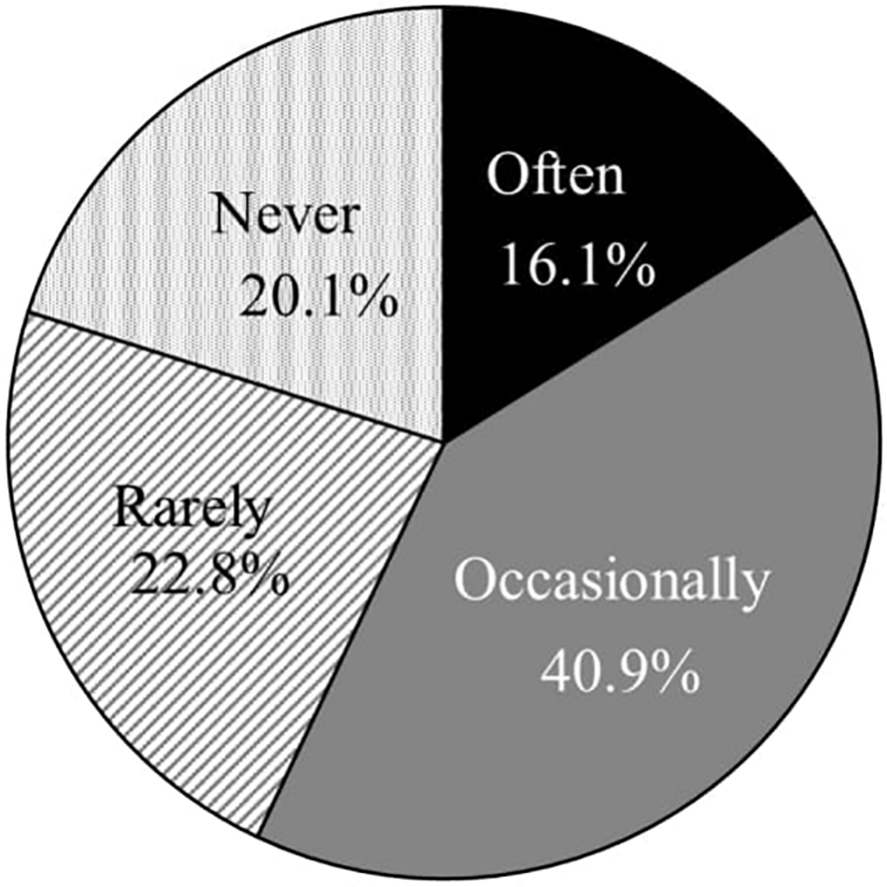
The frequency of missed medications (n = 149).

**Figure 2:**
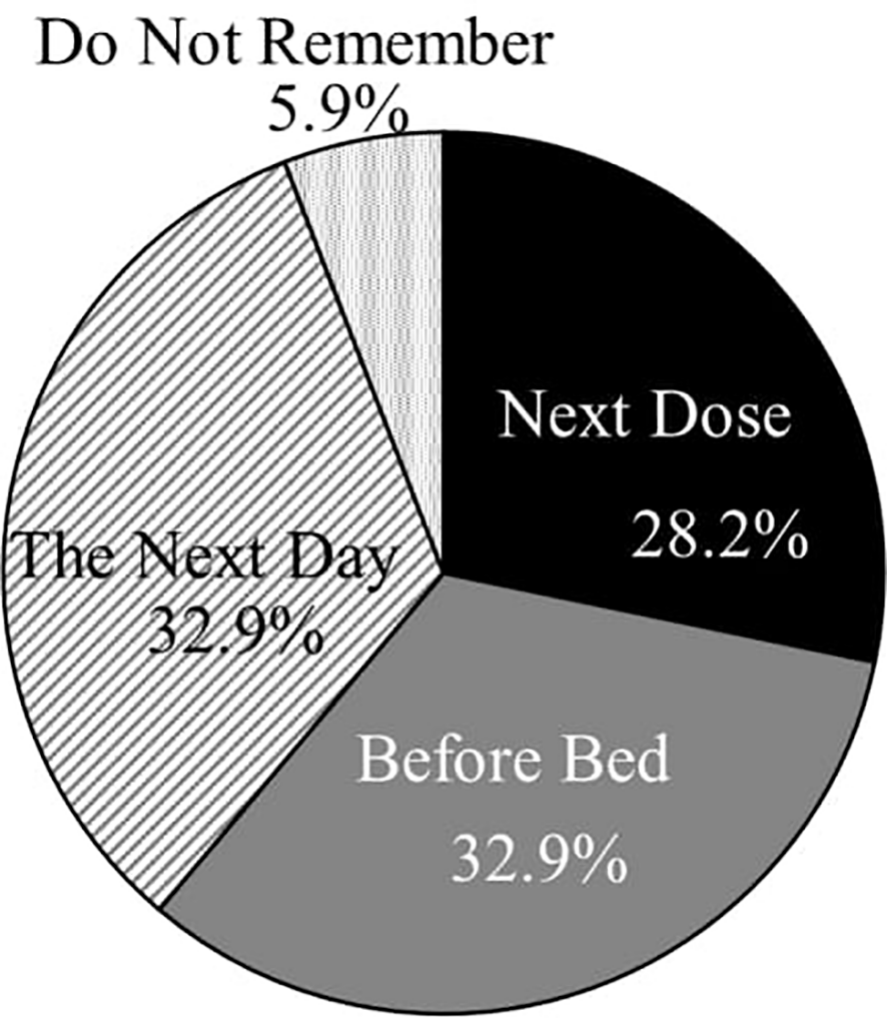
The timing of remembering missed medications (n = 85).

**Figure 3:**
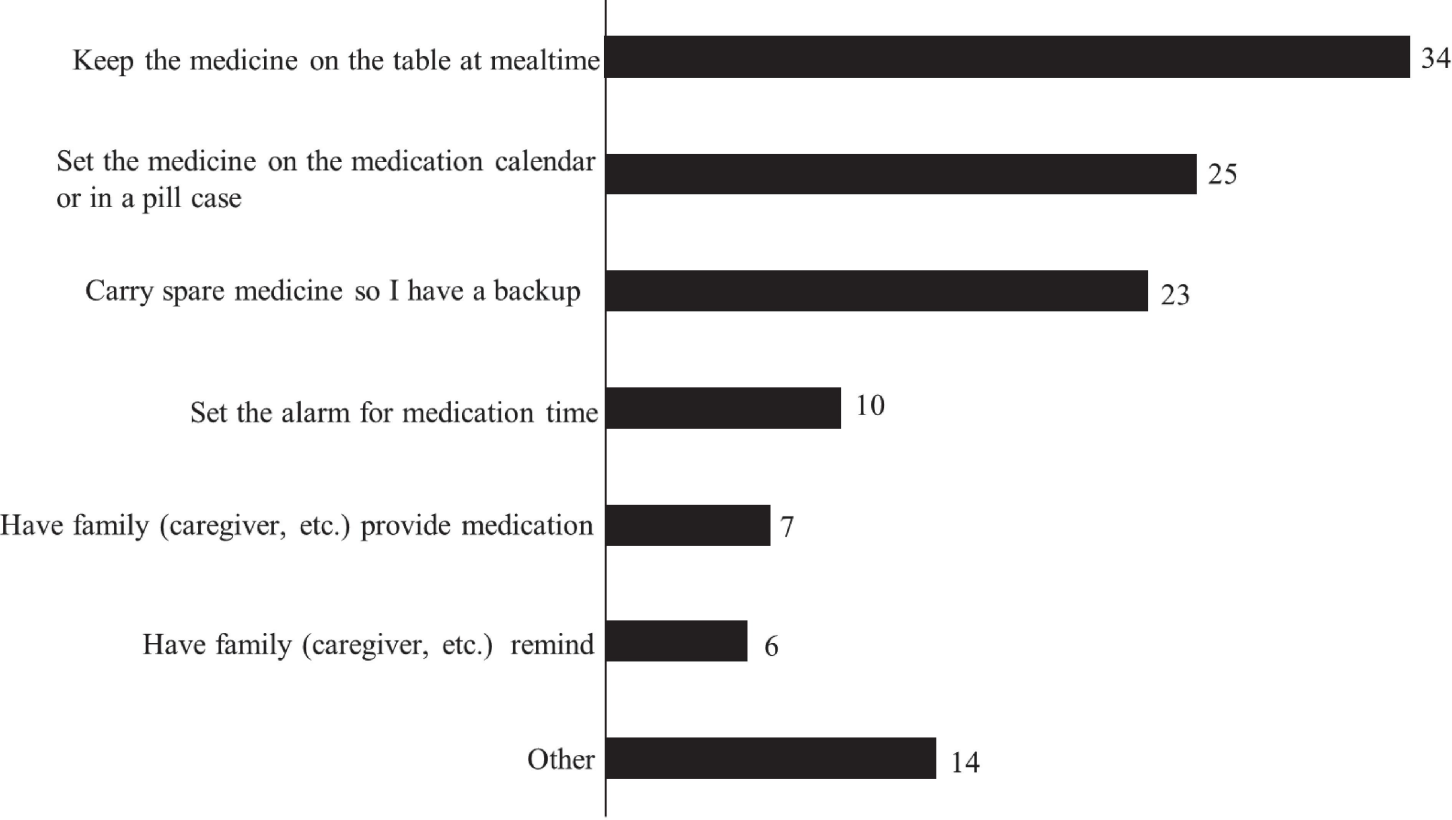
Specific measures (multiple choice) (n = 72). Seventy-two (72) patients reported employing strategies to prevent forgetting their medication. We asked them to select multiple choice regarding specific measures.

**Figures 1** and **2** illustrate the frequency of missed doses and the timing of recollections of missed doses. Of the 149 patients, 48.3% (72) reported employing strategies to prevent forgetting their medication. The most prevalent method was placing the medication on the dining table during meals, followed by organizing medications on a calendar or in a pill case and carrying an extra dose to avoid oversights (**Figure 3**). The responses of others included “one-dose packages,” “setting reminders,” “writing the date,” “placing the medicine on the table before going to sleep,” and “putting a reminder on the computer.” Despite these efforts, 65.3% (47 out of 72) of these patients still frequently forgot to take their medication.

### 3.4 Frequency of missed medication

A total of 149 participants in this study were taking medication.

**Figure 4:**
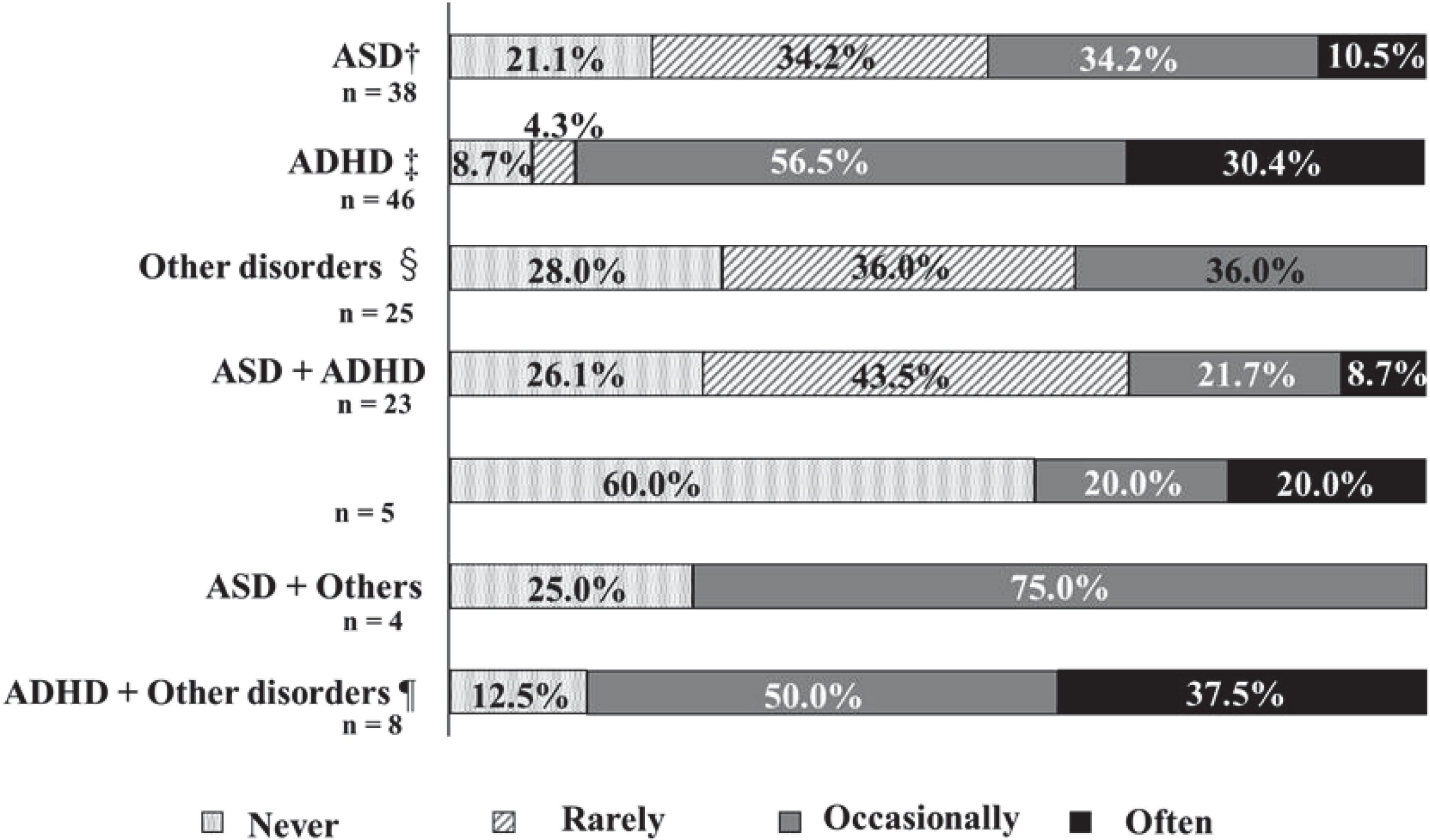
The frequency of missed medication and disorders. †ASD: Autism Spectrum Disorders. ‡ADHD: Attention Deficit Hyperactivity Disorder. §Other disorders: Learning Disability (LD), Stuttering, Others. ¶Other disorders: LD, Tic disorders, Others. The percentage of patients who reported forgetting their medication "often" or "occasionally" was 86.9% for patients with ADHD, and 87.5% for patients with ADHD and other disorders.

**Figure 1** illustrates the frequency of medication non-adherence, while **Figure 4** details the percentages by disease. The percentage of patients who reported forgetting their medication “often” or “occasionally” was 86.9% for patients with ADHD, and 87.5% for patients with ADHD and other disorders.

**Figure 5:**
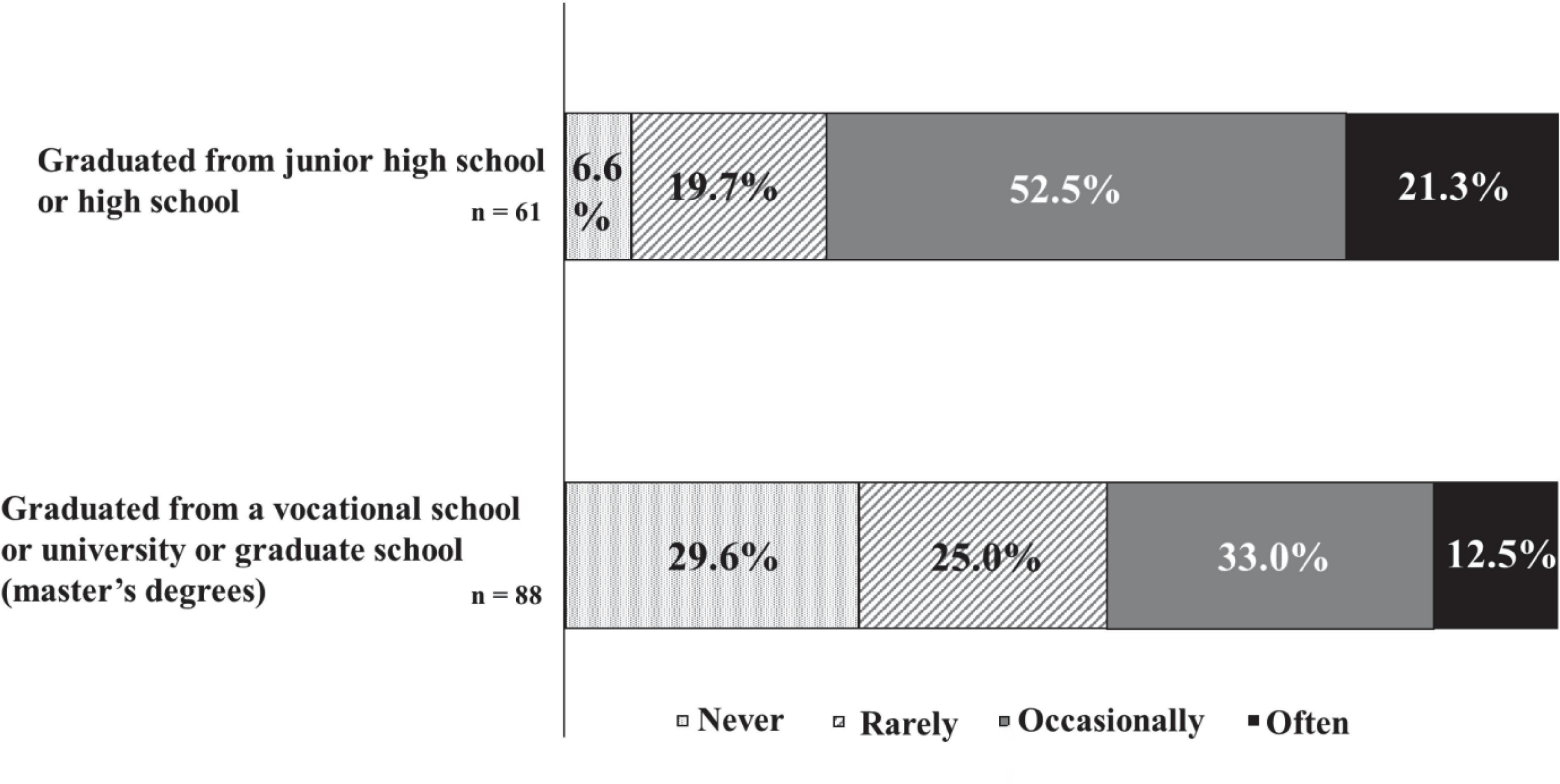
The frequency of missed medication and final education. There were 73.8% patients with junior high or high school education reported forgetting their medication "often" or "occasionally".

**Figure 5** reveals that patients with junior high or high school education reported forgetting their medication “often” or “occasionally” more frequently than those with vocational school, university, or graduate school education.

**Figure 6:**
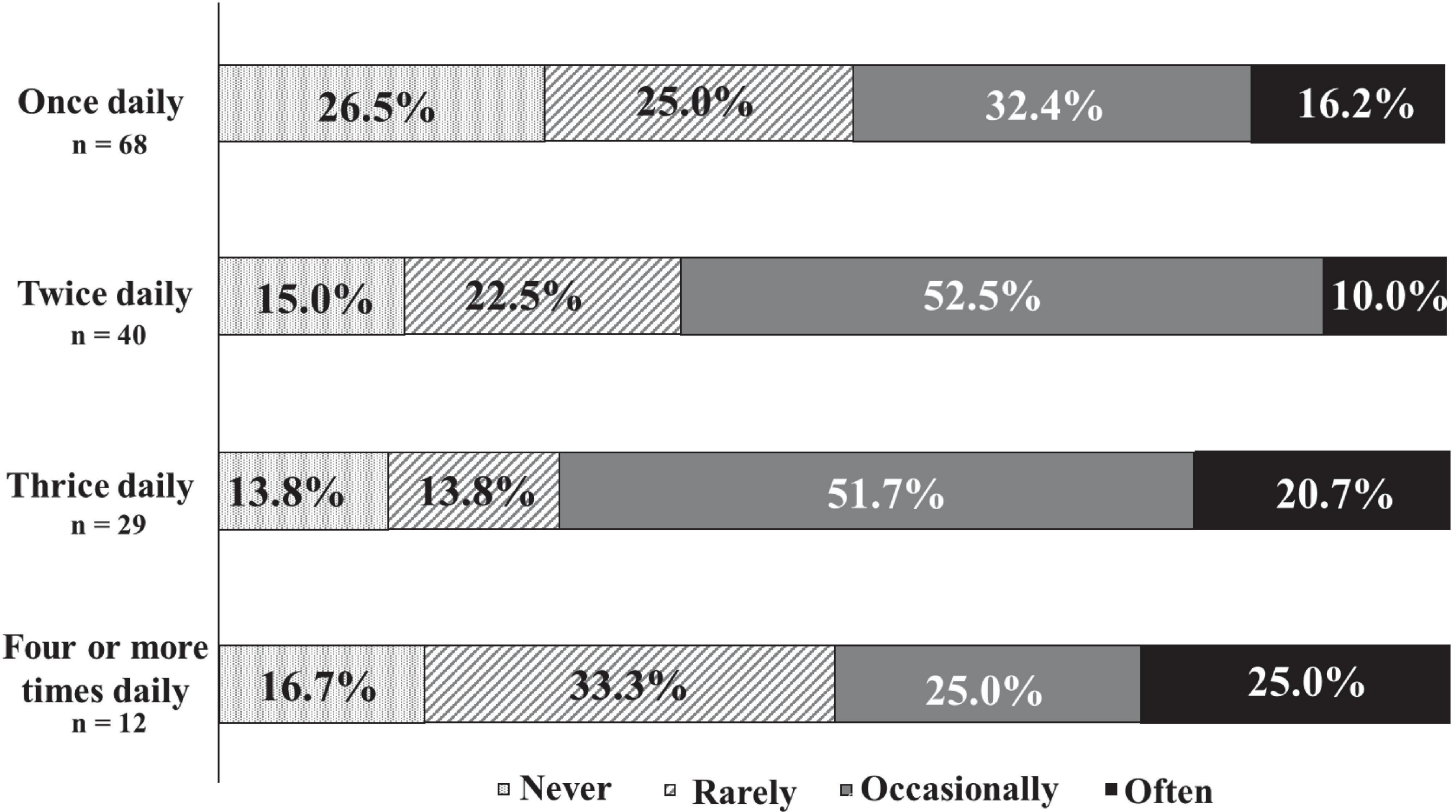
The frequency of missed medication and number of doses. There were 72.4% of patients who took medication three times daily experienced the highest frequency of missed doses.

**Figure 6** indicates that patients who took medication three times daily experienced the highest frequency of missed doses.

### 3.5 Current status regarding medication record books

Among the 198 participants, 85.4% (169) owned a paper medication record book, 6.1% (12) had a digital version on a smartphone, 4.5% (9) maintained both paper and digital versions, and 4.0% (8) did not own any medication record book. Of the 190 patients who either or both paper or digital versions of their medication record books, 59.5% (113) reported that they almost always brought their medication record books to medical appointments, 19.5% (37) often brought them, 12.1% (23) frequently did not, and 8.9% (17) never brought them. Regarding usage, 23.2% (44 out of 190) strongly agreed, 38.4% (73 out of 190) somewhat agreed, 30.0% (57 out of 190) did not strongly agree, and 8.4% (16 out of 190) disagreed that they effectively used their medication record books. The primary reasons for utilizing the medication record book included sharing prescription details with healthcare providers (82 patients) and checking the prescription details themselves (73 patients). However, 41 patients did not voluntarily consult the medication record book, and another 73 found it unnecessary to do so at every visit.

## 4 Discussion

This study utilized a sample of 200 patients who were recruited from the Showa University Research Institute for Developmental Disabilities. The mean age of the patients was 33 years, which distinguishes this study as a unique investigation of adults. A significant proportion of the patients in this study experienced some degree of difficulty in their daily lives and were treated on an outpatient basis. Nevertheless, given their high level of education and previous attendance at vocational, university, and graduate schools, it is reasonable to conclude that those who participated in this study possess the capacity to comprehend the information provided by medical professionals, including doctors and pharmacists. Nevertheless, this study found that 57.0% of the participants with DD were non-adherent to their medication regimens. Medication non-adherence remains a significant challenge in this population. The adherence rate to psychotropic medications among patients with DD and psychiatric disorders is reported at only 25.7% (27). For individuals with ADHD, non-adherence rates range from 13.2% to 65% (19, 20). Our findings indicate that the frequency of medication non-adherence among patients with DD in Japan aligns with rates observed in other countries. However, existing literature presents inconclusive results due to significant individual variability (19).

The causes of non-adherence to ADHD medications are multifaceted, encompassing both patient- and drug-related factors (28). Patient-related factors such as disorganization and forgetfulness complicate adherence to traditional dosing schedules that require multiple daily doses (10, 28). Consequently, inadequate supervision of medication administration can result in missed doses. Drug-related issues include the necessity of multiple daily doses to maintain efficacy (28). The most frequently taken medications ranged from one to three. In this study, the rate of adherence was found to decrease as the number of medications taken increased, a result that was found to be consistent with those of other studies. Prescribing effective once-daily medications may mitigate these challenges. Additionally, societal attitudes and pressures also play roles in influencing medication adherence (19). Clinical experience suggests that some individuals feel embarrassed at having to take medication in public, rather than at home in private, and that there is a social stigma attached to having a psychiatric condition requiring treatment (19).

Discontinuing medication can escalate behavioral problems, anger, frustration, and adversely affect life at home, work, and school (21). Enhanced medication adherence not only manages the disease but also improving the adverse effects of stopping medication and helps prevent the development of comorbid conditions associated with DD (29).

Approximately 40% of adults have a psychiatric disorder, with 73.8% of these individuals reporting a history of such disorders (30). It is crucial to prioritize medication adherence among patients with these conditions. Notably, patients with ASD who also have ADHD demonstrate higher adherence to ADHD medications (31). Psychiatric disorders can significantly impact medication adherence in individuals with DD (32). Twenty-four percent (48) of the participants in this study also had other psychiatric comorbidities. This study observed non-adherence rates of 44.7% in individuals with ASD, 86.9% in those with ADHD, and 30.4% in patients with comorbid ASD and ADHD. As previously reported, medication adherence was higher among ASD patients with comorbid ADHD compared to ASD patients in this study. Additionally, the present study found higher medication adherence among ADHD patients with comorbid ASD compared to ADHD patients. Further research is necessary to ascertain the prevalence and impact of comorbidities in this population.

As noted in prior research, patient-related factors critically influence medication adherence (28). Treatments may include antidepressants, mood stabilizers, anxiolytics, antipsychotics, or stimulants if ADHD coexists with mood, anxiety, or psychotic disorders. Moreover, α-2 agonists are effective in treating ADHD symptoms, while certain psychotropic medications address aggressive, self-injurious, and stereotypic behaviors (17). Opioid antagonists may also be beneficial for managing self-injurious and stereotypic behaviors. This study, however, did not analyze specific medication types or dosing frequencies per disease.

The World Health Organization’s descriptive epidemiological study on ADHD in adults showed a notably higher prevalence of ADHD among individuals without a university education compared to university graduates (5). Conversely, this study found a higher likelihood of vocational school, university, or master’s degree attainment among participants compared to those with only junior high or high school education. This disparity may stem from the fact that many patients attending the Institute for Medical Research on Developmental Disorders are adults, which influences their reasons for seeking consultation. Indeed, a significant number of study participants reported that their age of onset was 18 years or older. Our hypothesis posited that lower levels of education would correlate with a reduced understanding of the disease and medication instructions, thereby impacting adherence. The findings revealed that participants with DD who had only attained junior high or high school education demonstrated higher rates of non-adherence compared to those with vocational school, university, or master’s degrees. Previous studies have noted that many patients in the pervasive developmental disorder group, first visiting the institute at age 18 or older, possessed at least a high school education and exhibited superior language comprehension (33). It is speculated that these individuals’ advanced language skills may have masked any related issues during their school-age years (33). Thus, this analysis suggests that lower educational levels might contribute to reduced adherence among patients with DD, supporting our initial hypothesis.

This study also explored the relationship between dosing frequency and patient adherence. Medication adherence worsened as the number of times the medication was taken increased from once a day to twice a day to three times a day. This is consistent with previous reports, suggesting that the same tendency may be observed in patients with DD. Other research indicates that adherence among older adults declines when medication is administered three or more times daily (26). However, in our study, half of the patients who took their medication four or more times daily reported forgetting to take it, showing an improvement in adherence compared to those taking medication three times daily. To date, no studies have confirmed that increased medication frequency enhances adherence. The uniqueness of these findings to this population or differences in patient attitudes toward frequent medication remains uncertain. We warrant further investigation given the limited sample size (n = 12) of patients taking more than four medications daily.

Continuing medication is vital for controlling disease, especially in patients with DD. Internationally, interventions like combination tablets, pharmacist consultations to enhance disease understanding, and medication reminders (e.g., via telephone) have proven effective (34). However, options for psychiatric combination tablets are limited in Japan. In this study, only a few patients opted for medication reminders, alarms, or family member assistance (e.g., caregivers), while many preferred visual measures. Notwithstanding, 65.3% of those using such interventions still experienced non-adherence, underscoring the need for pharmacist involvement in disease management. Consequently, pharmacists should offer guidance to implement effective strategies. In this study, 190 patients (96.0%) owned either a paper or smartphone medication record book, reflecting an increase from the 88.5% reported in a 2016 survey (35). Although medication record books are designed to facilitate information sharing between healthcare professionals and patients in Japan, they were not fully utilized. Given the primary role of medication in treating many psychiatric disorders and the growing global burden of these conditions, pharmacists are expected to play a more significant role in supporting individuals with psychiatric disorders.

Furthermore, this study examined the current status of medication record books. Of the 190 patients who possessed a medication record book, 59.5% almost invariably brought it with them to their office visits, and more than 60% of the participants reported that they utilized their medication record book with considerable frequency. Furthermore, the respondents indicated that the purpose of bringing a medication record book was to share prescription details with their healthcare provider. It was thus determined that DD patients utilize their medication record books as a means of facilitating communication with their healthcare providers. Furthermore, by checking the medication record book themselves, they are attempting to utilize it for their own medication management. These findings suggest that while the medication record book remains a valuable tool, its usage and perceived importance vary significantly between individuals. This highlights the necessity for a more nuanced understanding of patients’ needs and usage patterns, with tailored support being a crucial aspect of future medication support initiatives. Additionally, as digital technologies continue to evolve, the dissemination of digital versions of medication record books and their potential applications will be a crucial area of future research. This study represents the first investigation into the actual medication use among individuals with DD in Japan. The results confirm that medication non-adherence is a common issue both in Japan and Western countries. In addition, this study suggests that lower educational levels might contribute to reduced adherence among patients with DD. For adult patients with DD, it is essential that pharmacists provide not only guidance as patients with DD, but also intervention tailored to the individual, such as educational background and age of onset. For example, the spread of pharmacist outpatient care in psychiatry will help improve medication adherence. Moreover, this study showed that medication adherence worsened as the number of times the medication was taken increased from once a day to twice a day to three times a day. Therefore, it is recommended that dosage forms be proposed to reduce the number of times the medication is taken, and that new drugs, such as LAIs, patches, and combination drugs, be developed. Although medication record books hold potential for enhancing disease management, their full potential has not yet been realized, indicating a need for greater utilization.

## 5 Limitation

This study has some limitations. First, this study was a self-administered questionnaire, so it was not possible to ask and collect detailed information that may affect medication adherence. Second, there is no number or percentage that determines whether medication adherence is good in Japan. In other words, medication adherence cannot be quantified, and evaluation is based on the patient’s subjective assessment of the degree of difficulty and the effectiveness of measures based on that.

## 6 Conclusion

The study indicates that the frequency of medication non-adherence among patients with DD in Japan mirrors that in other countries. Patients who reported taking preventative measures still experienced high rates of non-adherence, suggesting limited effectiveness of these strategies. It is essential to develop more effective measures to improve adherence, enhance disease awareness, and increase understanding of medication instructions. The high possession rate of medication record books suggests they could play a significant role in managing DD, and their use is expected to increase in the future.

## Data Availability

The datasets presented in this article are not readily available because Due to the nature of this research, participants of this study did not agree for their data to be shared publicly, so supporting data is not available. Requests to access the datasets should be directed to Noriko Hida, n.hida@med.showa-u.ac.jp.
